# Supplementary material for: Transdermal electroosmotic flow generated by a porous microneedle array patch
Source: Nat Commun. 2021 Jan 28;12:658. doi: 10.1038/s41467-021-20948-4 (PMC7843990; doi:10.1038/s41467-021-20948-4)
Supplement: Supplementary file 1 — Supplementary Information [file 41467_2021_20948_MOESM1_ESM.pdf]

Supplementary Information

Transdermal electroosmotic flow generated by a porous microneedle array patch

Shinya Kusama[1], Kaito Sato[1], Yuuya Matsui[1], Natsumi Kimura[1], Hiroya Abe[1], Shotaro Yoshida[1] & Matsuhiko Nishizawa[1,2]*

[1] Department of Finemechanics, Graduate School of Engineering, Tohoku University, 6-6-1 Aramaki Aoba, Aoba-ku, Sendai 980-8579, Japan

[2] Division for the Establishment of Frontier Sciences of the Organization for Advanced Studies, Tohoku University, 2-1-1 Katahira, Aoba-ku, Sendai, 980-8577, Japan

*email: nishizawa@biomems.mech.tohoku.ac.jp.

Supplementary Figure 1 ~ Figure 13

Supplementary Table 1 and Table 2

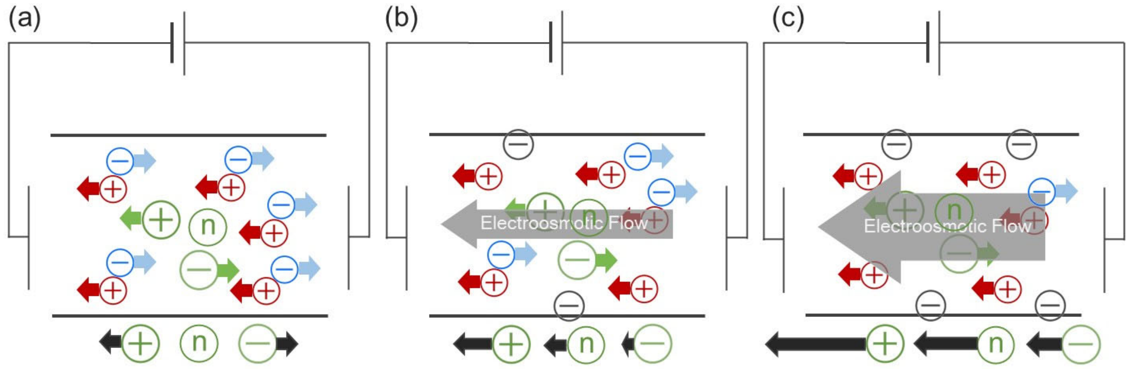

Supplementary Figure 1. Iontophoresis consists of the electrophoretic motion of the charged molecules themselves as well as the electroosmotic flow (EOF) generated by the fixed charges. (a) Electrophoresis of the positively charged, neutral and negatively charged molecules (green) without EOF. (b, c) The net motion of the molecules with the EOF generated by the preferential movement of mobile cations, where the smaller (b) and larger (c) amount of negative charges are fixed (gray).

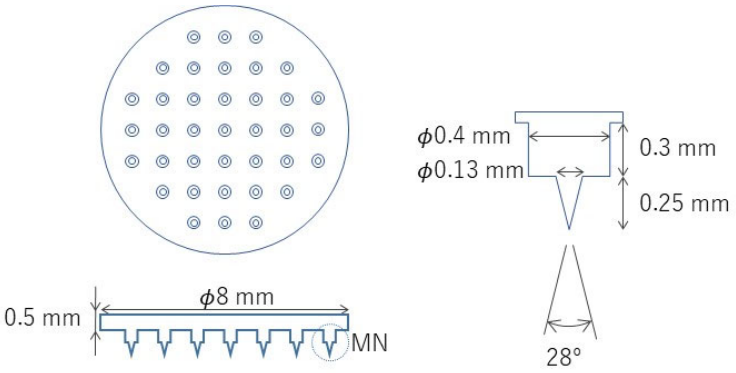

Supplementary Figure 2. The detailed geometry of the PMN array chip.

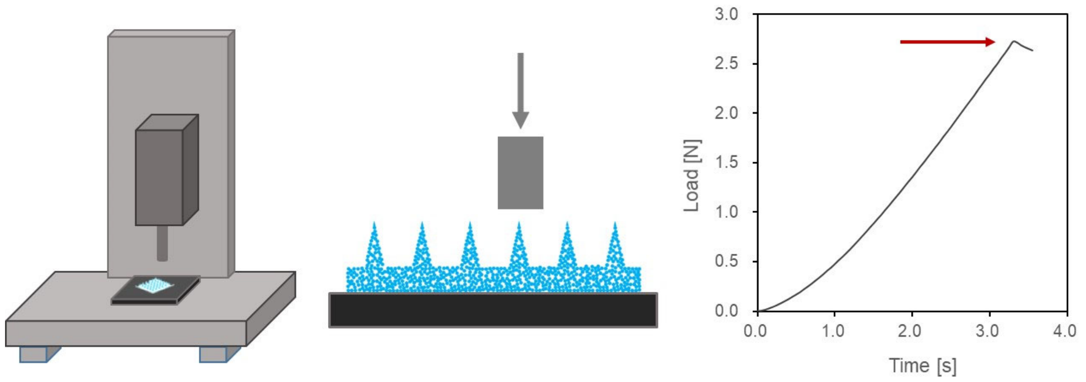

Supplementary Figure 3. The measurement of the compression fracture force of a porous needle. A metal jig was pushed from above onto the tip of a microneedle at a rate of 4 mm/min. The force on the metal jig and displacement were simultaneously recorded by a force gauge, and fracture force was defined as the maximum force before the sharp drop of force. A fracture force of 2.5 N ± 0.26 (N = 3 independent experiments; mean ± SD) was obtained.

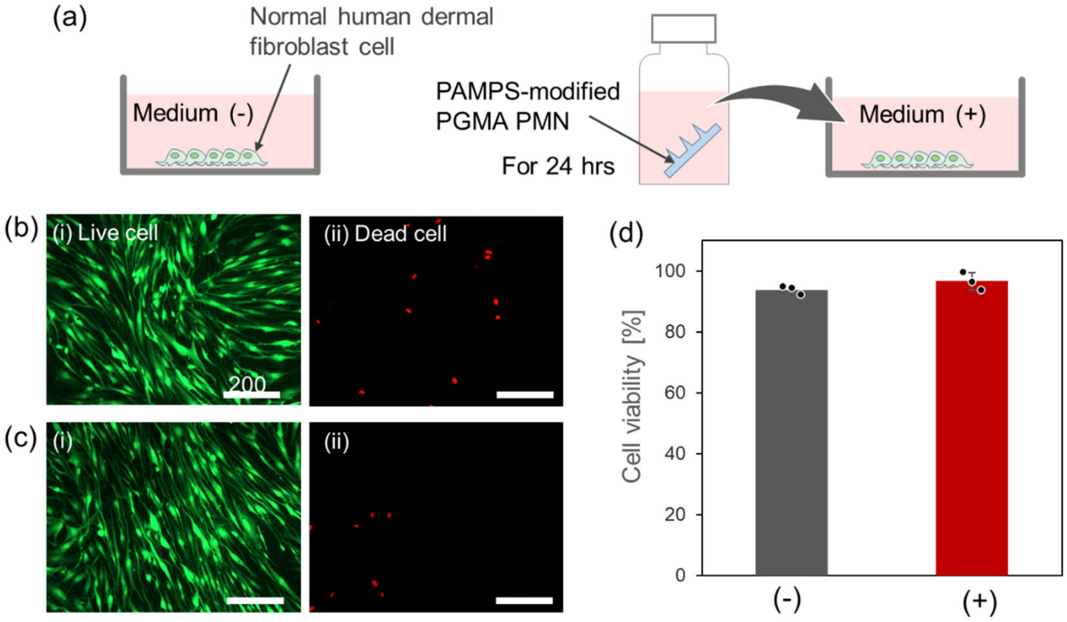

Supplementary Figure 4. Biocompatibility of the PAMPS-modified PGMA PMN. (a) Illustration showing normal human dermal fibroblasts (NHDF) with a density of 4.71×10^4 cells /cm^2 seeded to a 12-well dish, and cultured for 3 days in two DMEM medias. (b) The Live/Dead staining of the cells cultured with the DMEM medium without presoaking PMN. (c) The Live/Dead staining of the cells cultured with the DMEM medium prepared by presoaking PMN for 24 hours. (d) Cell viability derived using ImageJ for the cases with (+) and without (-) the presoaking of the PMN (N = 3 independent experiments; mean ± SD).

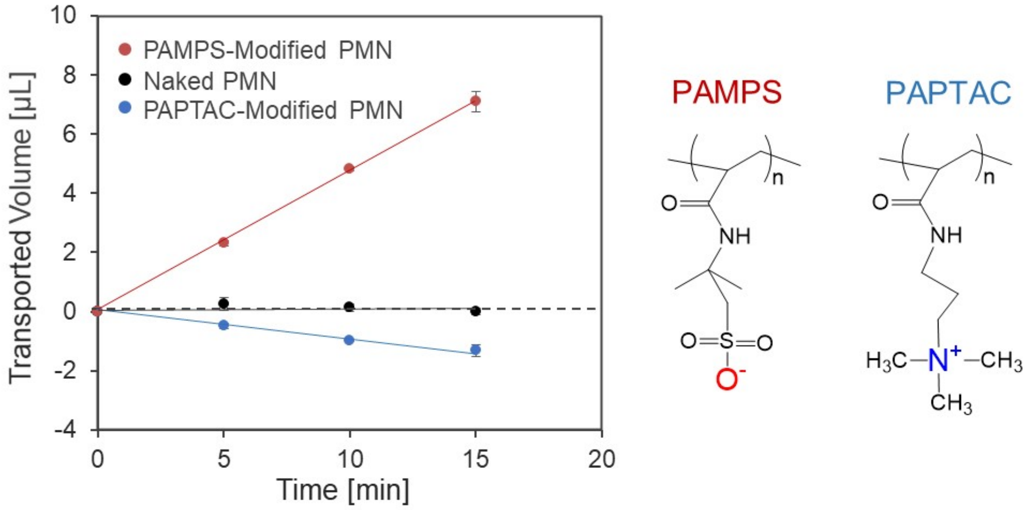

Supplementary Figure 5. The accumulated amount of water transported at 1 mA / cm^2 through the PAMPS-filled PMN prepared from 1.5 M AMPS (red), the naked PMN (black) and the PAPTAC-filled PMN prepared from 1.5 M (3-acrylamidopropyl) trimethylammonium chloride (APTAC) (blue). pH7 McIlvaine buffer was used. Error bars indicate standard error of mean (N = 3 independent samples; mean ± SD). The preliminary result obtained by the modification of PAPTAC showed the water flow of opposite direction. The flow rate, which was slower than the case of PAMPS, will be improved by optimization of the polymerization condition of PAPTAC.

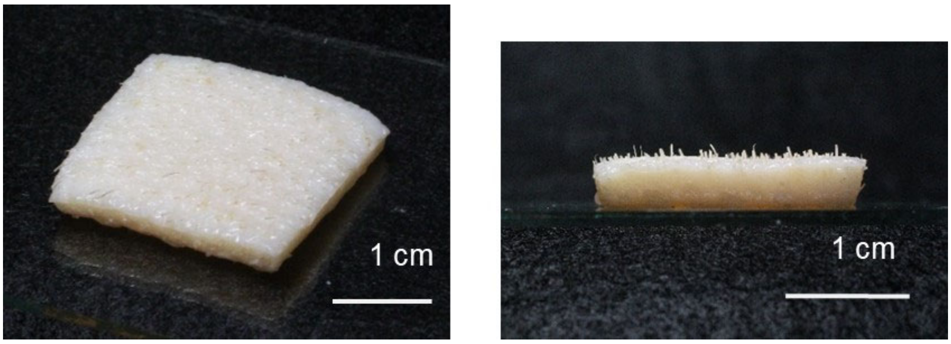

Supplementary Figure 6. The pictures of a piece of pig abdominal skin used for experiments.

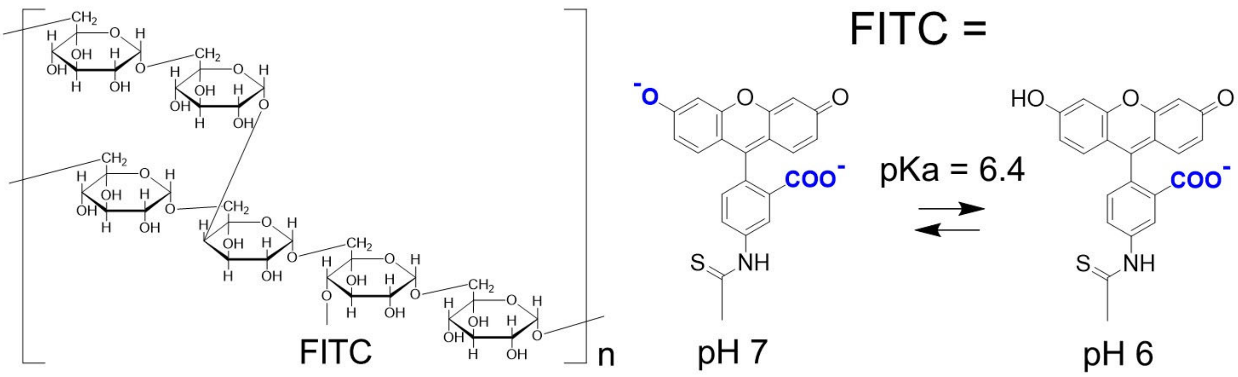

Supplementary Figure 7. The chemical structure of FITC-dextran molecule with the protonation / deprotonation on the FITC moiety at different pH.

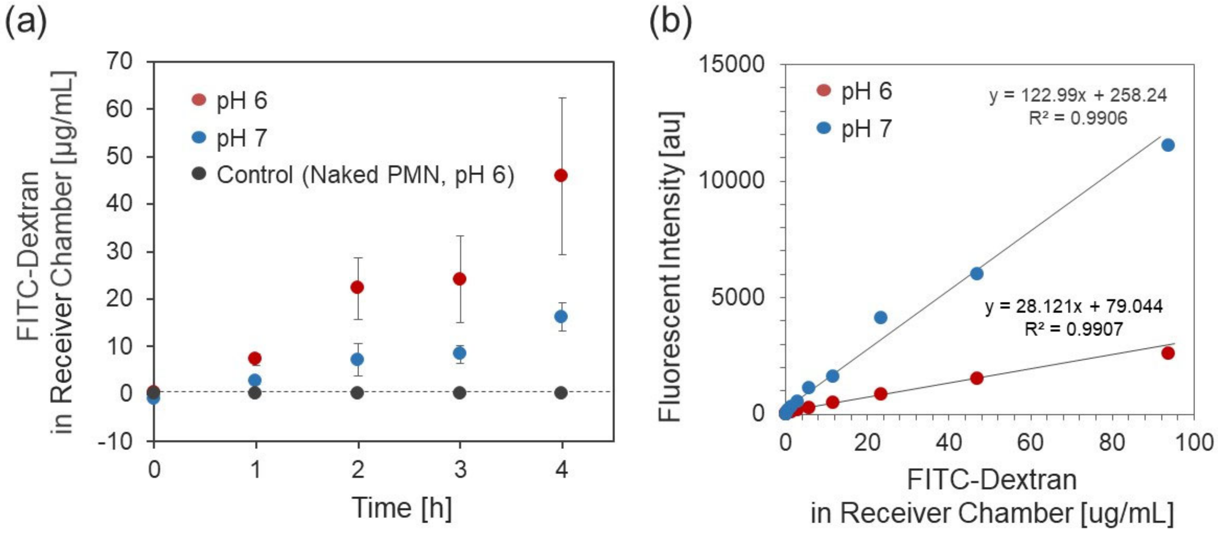

Supplementary Figure 8. (a) The time-course of the accumulated amount of FITC-dextran transported to the receiver chamber through the naked PMN (black) and the PAMPS-modified PMN at pH 6 (red) and pH 7 (blue) during application of 3 mA/cm^2 (N = 3 independent experiments; mean ± SD). (b) The calibration curves used for the calculation of the amount of FITC-dextran in the receiver chamber from the fluorescent intensity at 520 nm.

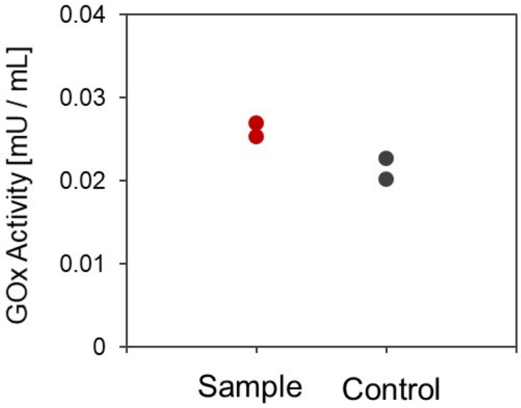

Supplementary Figure 9. The catalytic activity of the glucose oxidase (100 mg/mL Gox, 150 kDa, Toyobo) before (black) and after (red) the application of electroosmotic flow at 0.5 mA/cm^2 for 42 hours. The catalytic activity was measured by using an absorptiometer (SEC 2020, ALS) and a GOx activity assay kit (MAK097, Sigma Aldrich).

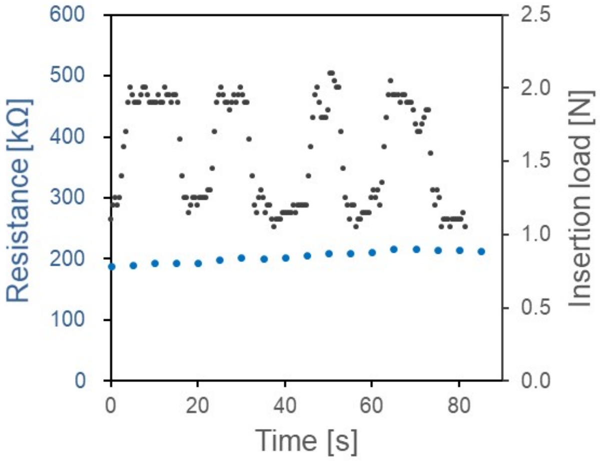

Supplementary Figure 10. The trans-skin resistance measured under varying insertion pressure (1.0 ~ 2.0 N) applied to the PMN on human arm skin.

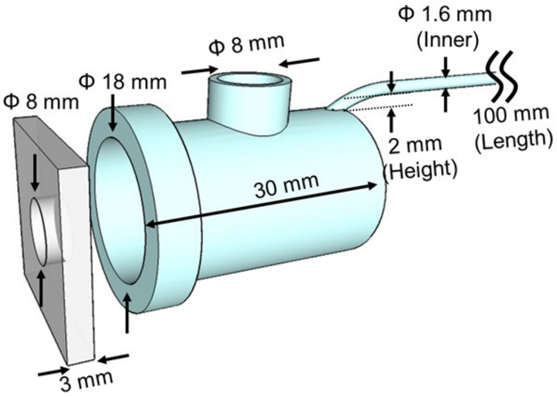

Supplementary Figure 11. The dimensions of the side-by-side Franz cells with a horizontal capillary, manufactured for evaluation of water flow during DC current application.

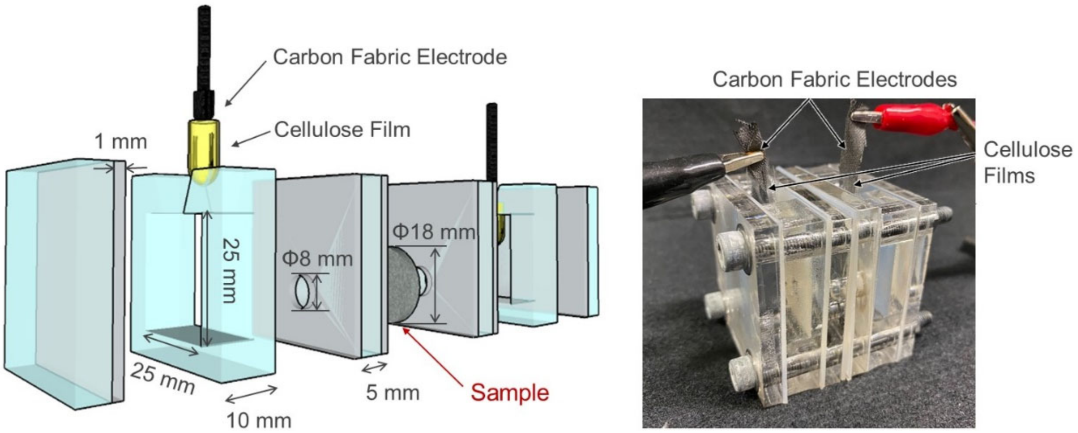

Supplementary Figure 12. The illustrated structure and a photograph of the Frantz cell used for evaluation of molecular transport through skins and PMNs. The parts of the cell were handmade with acrylic plates and silicone sheets. The electrodes for current application were the carbon fabrics (CFs) covered by the cellulose semipermeable membranes to prevent the effects of electrolysis.

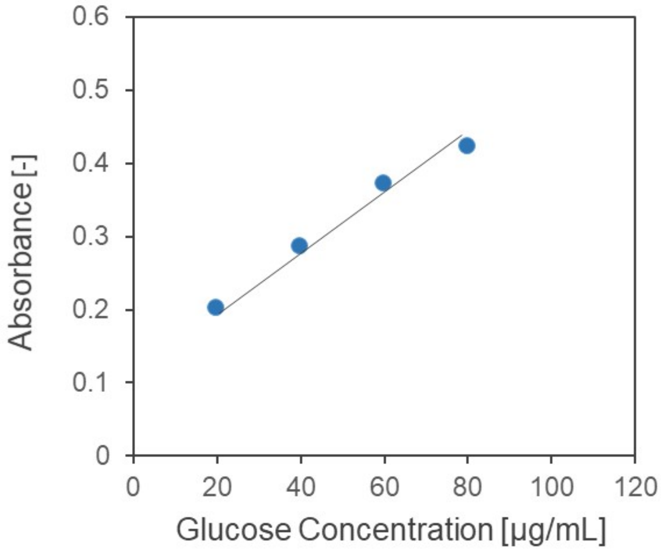

Supplementary Figure 13. The calibration curve used for calculation of the extracted glucose, which was prepared by experiments according to the protocol of the commercialized enzyme colorimetric assay kit (GAGO20, Sigma Aldrich).

Supplementary Table 1 Classification and Characteristics of Microneedles (MNs)

Type of MN		Material	Fabrication	Precision in Miniaturization	Mechanical Strength	Transparency	Permeability	Flow Mechanism	Reference Papers
Conventional Solid MN	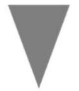	Metal, Oxide, Polymer	Molding Photolithography	High	High	+			26, 27
Dissolvable MN	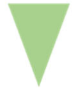	Biomaterials	Molding	High	Low	+			28-31
Hydrogel MN	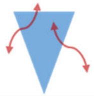	Xerogel (Dried Hydrogel)	Molding	High	Low	+	Need Swelling		32-34
Hollow MN	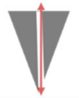	Metal, Oxide Polymer	Laser Processing Photolithography	Low	High	+	Partly	Pressure Flow	35-37
Surface Porous MN	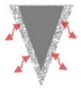	Metal, Oxide Polymer	Molding	High	High	-			38-40
Porous MN	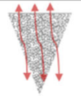	Metal, Oxide, Polymer	Molding	High	Low	-	Entirely	Pressure Flow	41-45
Charge-Modified Porous MN	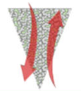	Polymer	Molding	High	Low	-	Entirely	EOF	This Study

Gray cells indicate drawbacks.

Supplementary Table 2 Composition of Electrolyte Solutions Used

	Ringer's solution	147.2 mM NaCl, 4.02 mM KCl, 2.24 mM $CaCl_2$	for evaluating DC resistance
pH 5	McIlvaine buffer	103 mM Na_2HPO_4, 48.5 mM Citric acid	for preparing FDH anode
pH 6	McIlvaine buffer	126.3 mM Na_2HPO_4, 36.85 mM Citric acid	for experiments of FITC-dextran transport
pH 7	McIlvaine buffer	164.7 mM Na_2HPO_4, 17.65 mM Citric acid	for all other experiments
