# Supplementary material for: Transdermal electroosmotic flow generated by a porous microneedle array patch
Source: Nat Commun. 2021 Jan 28;12:658. doi: 10.1038/s41467-021-20948-4 (PMC7843990; doi:10.1038/s41467-021-20948-4)
Supplement: Supplementary file 3 — Reporting Summary [file 41467_2021_20948_MOESM3_ESM.pdf]

nature research

Corresponding author(s): Matsuhiko Nishizawa

Last updated by author(s): Nov 20, 2020

Reporting Summary

Nature Research wishes to improve the reproducibility of the work that we publish. This form provides structure for consistency and transparency in reporting. For further information on Nature Research policies, see our Editorial Policies and the Editorial Policy Checklist.

Statistics

For all statistical analyses, confirm that the following items are present in the figure legend, table legend, main text, or Methods section.

n/a	Confirmed	
☐	☒	The exact sample size (*n*) for each experimental group/condition, given as a discrete number and unit of measurement
☐	☒	A statement on whether measurements were taken from distinct samples or whether the same sample was measured repeatedly
☐	☒	The statistical test(s) used AND whether they are one- or two-sided<br>*Only common tests should be described solely by name; describe more complex techniques in the Methods section.*
☐	☒	A description of all covariates tested
☐	☒	A description of any assumptions or corrections, such as tests of normality and adjustment for multiple comparisons
☐	☒	A full description of the statistical parameters including central tendency (e.g. means) or other basic estimates (e.g. regression coefficient) AND variation (e.g. standard deviation) or associated estimates of uncertainty (e.g. confidence intervals)
☒	☐	For null hypothesis testing, the test statistic (e.g. *F*, *t*, *r*) with confidence intervals, effect sizes, degrees of freedom and *P* value noted<br>*Give P values as exact values whenever suitable.*
☒	☐	For Bayesian analysis, information on the choice of priors and Markov chain Monte Carlo settings
☒	☐	For hierarchical and complex designs, identification of the appropriate level for tests and full reporting of outcomes
☒	☐	Estimates of effect sizes (e.g. Cohen's *d*, Pearson's *r*), indicating how they were calculated

Our web collection on statistics for biologists contains articles on many of the points above.

Software and code

Policy information about availability of computer code

Data collection	No software was used.
Data analysis	ImageJ (1.52q)

For manuscripts utilizing custom algorithms or software that are central to the research but not yet described in published literature, software must be made available to editors and reviewers. We strongly encourage code deposition in a community repository (e.g. GitHub). See the Nature Research guidelines for submitting code & software for further information.

Data

Policy information about availability of data

All manuscripts must include a data availability statement. This statement should provide the following information, where applicable:

- Accession codes, unique identifiers, or web links for publicly available datasets
- A list of figures that have associated raw data
- A description of any restrictions on data availability

https://doi.org/10.6084/m9.figshare.13264922 (Link before publish: https://figshare.com/s/ef2a2686fee9131bba6c)

Field-specific reporting

Life sciences study design

All studies must disclose on these points even when the disclosure is negative.

Sample size	The size of PMN chip used for the measurement of DC resistance of human skin arm (N = 6, independent measurements for 3 human subjects) was determined by the size of the holes in the permeation cell used for the studies of pig skin sample for comparison.
Data exclusions	No data were excluded from the analysis.
Replication	We conducted independent experiments 6 times (N = 6, independent experiments for 3 human subjects), and all attempts at replication were successful.
Randomization	All allocation was random.
Blinding	All investigators were blinded for data analysis.

Reporting for specific materials, systems and methods

We require information from authors about some types of materials, experimental systems and methods used in many studies. Here, indicate whether each material, system or method listed is relevant to your study. If you are not sure if a list item applies to your research, read the appropriate section before selecting a response.

Materials & experimental systems

n/a	Involved in the study	
☒	☐	Antibodies
☒	☐	Eukaryotic cell lines
☒	☐	Palaeontology and archaeology
☒	☐	Animals and other organisms
☐	☒	Human research participants
☒	☐	Clinical data
☒	☐	Dual use research of concern

Methods

n/a	Involved in the study	
☒	☐	ChIP-seq
☒	☐	Flow cytometry
☒	☐	MRI-based neuroimaging

Human research participants

Policy information about studies involving human research participants

Population characteristics	3 student participants (25 years-old male, 24 years-old male, 24 years-old female)
Recruitment	They were recruited in the laboratory of the corresponding author.
Ethics oversight	All procedures performed in studies involving human participants were in accordance with the standards of Ethics Committee of Graduate School of Engineering, Tohoku University (16A-4).

Note that full information on the approval of the study protocol must also be provided in the manuscript.
